# Effects of diurnal, lighting, and angle-of-incidence variation on anterior segment optical coherence tomography (AS-OCT) angle metrics

**DOI:** 10.1186/s12886-017-0425-3

**Published:** 2017-03-23

**Authors:** Handan Akil, Anna Dastiridou, Kenneth Marion, Brian A. Francis, Vikas Chopra

**Affiliations:** 1 Doheny Image Reading Center, Doheny Eye Institute, 1355 San Pablo Street, Los Angeles, CA 90033 USA; 2 Department of Ophthalmology, David Geffen School of Medicine, Los Angeles, CA USA

**Keywords:** Anterior chamber angle, Anterior segment OCT, Diurnal variation, Angle metrics

## Abstract

**Background:**

First reported study to assess the effect of diurnal variation on anterior chamber angle measurements, as well as, to re-test the effects of lighting and angle-of-incidence variation on anterior chamber angle (ACA) measurements acquired by time-domain anterior segment optical coherence tomography (AS-OCT).

**Methods:**

A total of 30 eyes from 15 healthy, normal subjects underwent anterior chamber imaging using a Visante time-domain AS-OCT according to an IRB-approved protocol. For each eye, the inferior angle was imaged twice in the morning (8 am – 10 am) and then again in the afternoon (3 pm – 5 pm), under light meter-controlled conditions with ambient room lighting ‘ON’ and lights ‘OFF’, and at 5° angle of incidence increments. The ACA metrics measured for each eye were: angle opening distance (AOD, measured 500 and 750 μm anterior from scleral spur), the trabecular-iris-space area (TISA, measured 500 and 750 μm anterior from scleral spur), and scleral spur angle. Measurements were performed by masked, certified Reading Center graders using the Visante’s Internal Measurement Tool. Differences in measurements between morning and afternoon, lighting variations, and angle of incidence were compared.

**Results:**

Mean age of the participants was 31.2 years (range 23–58). Anterior chamber angle metrics did not differ significantly from morning to afternoon imaging, or when the angle of incidence was offset by 5° in either direction away from the inferior angle 6 o’clock position. (*p*-value 0.13-0.93). Angle metrics at the inferior corneal limbus, 6 o’clock position (IC270), with room lighting ‘OFF’, showed a significant decrease (*p* < 0.05) compared to room lighting ‘ON’.

**Conclusions:**

There does not appear to be significant diurnal variation in AS-OCT parameters in normal individuals, but lighting conditions need to be strictly controlled since variation in lighting led to significant variability in AS-OCT parameters. No changes in ACA parameters were noted by varying the angle-of-incidence, which gives confidence in being able to perform longitudinal studies in approximately the same area (plus/minus 5° of original scan location).

**Electronic supplementary material:**

The online version of this article (doi:10.1186/s12886-017-0425-3) contains supplementary material, which is available to authorized users.

## Background

Primary angle closure glaucoma (PACG) is one of the leading causes of irreversible visual morbidity in the world [1]. The mechanisms are largely connected to poor filtration of aqueous fluid through the anterior chamber angle (ACA) [2]. Evaluation and measurement of the ACA is relevant to risk assessment of angle closure [3]. Gonioscopy, ultrasound biomicroscopy (UBM) and the scheimpflug systems have been used for the visualization and measurement of the ACA [2–6]. Anterior segment optical coherence tomography (AS-OCT) is a newer non-contact method of imaging which affords the evaluation of the ACA [4, 5]. Certain types of AS-OCT machines, like Visante OCT (Carl Zeiss, Meditec, Dublin, CA, USA), utilize time-domain technology [6, 7], and. allow visualization and evaluation of irido-corneal boundaries and the angle configurations [8, 9]. OCT imaging of the anterior segment could advance detection of angle metrics and reveal anatomical insights into the pathophysiology of angle closure glaucoma [10]. The ACA measurements obtained by AS-OCT have helped much to elucidate the mechanisms of angle closure glaucoma in the management of patients in clinical practice [7, 11, 12].

Low light conditions have been known to be associated with acute attacks of angle closure. Patients with shallow anterior chamber, shorter axial length, and larger lens volume, are statistically demonstrated to have increased risk of angle closure. However, the majority of eyes with these anatomic conditions do not develop angle closure glaucoma [13]. Therefore recent studies have tried to investigate the role of dynamic factors, such as pupil dilation on angle measurements [14–16]. Furthermore intraocular pressure, as well as biometric variables, including axial length, central cornea thickness and anterior chamber depth and volume have been shown to demonstrate a diurnal flunctuation [17, 18]. Therefore, it is important to know whether the ACA also demonstrates a change throughout the day. Finally, previous research has also shown marked differences between the measurements of ACA in different quadrants of the eye [19, 20]. Based on these studies, one could hypothesize that there may be differences when the scan line is varied in the same quadrant. This study explored the effect of varying the scan line in the inferior quadrant near the 6 o’clock position of inferior corneal limbus (IC 270°) during the morning and afternoon. We previously demonstrated that Visante Time Domain (TD)-OCT has been repeatable and reproducible for measurement of the angle, allowing us to measure the ACA quantitatively and derive reasonable angle metrics [21].

The objectives of our present study was to first evaluate the changes in ACA metrics for a diurnal effect in asymptomatic normal eyes, second was to examine how the angle of incidence affects measurements, and third was to test the effect of physiologic dilation via room lighting on ACA metrics.

## Methods

Fifteen healthy volunteers were recruited for this study which followed the tenets of the Declaration of Helsinki, the Health Insurance Portability and Accountability Act and had the Institutional Review Board approval.

**Fig. 1:**
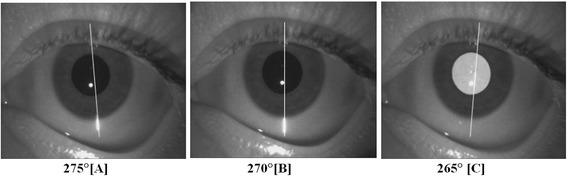
The set of images obtained with time-domain anterior segment optical coherence tomography at the IC275 (**a**), 270 (**b**), and 265 (**c**) positions

Each subject had both eyes (total of 30 eyes) imaged with the Visante TD-OCT (Carl Zeiss Meditec, Dublin, CA). The first set of images was obtained in the morning under standard room lighting at the IC270 position and also in a darkened room at the IC275, 270, and 265 positions (Fig. 1).

The light levels was read at the eye/camera interface with a Sper Scientific Light Meter (FC – 840021, Sper Scientific, Scottsdale, AZ). Each eye was let to adjust to the light level for 1 min in the room before imaging. Two different light levels were used; a light meter reading of 1.0 fc (fc;1 fc 10.77 cd/m^2^) (lights ON) and .0.0 fc (Lights OFF).

The varying angle of incidence was based on the visualization of the scan line by the OCT technician taking the images. The second set of images was obtained in the afternoon under the same conditions as the morning. The inferior angle opening distance (AOD, measured 500 and 750 μm anterior from Scleral Spur), trabecular-iris space area (TISA, measured 500 and 750 μm anterior from Scleral Spur), and scleral spur angle were computed for each eye and condition.

The Visante TD-OCT internal angle measurement tools were used to determine angle metrics based on the visualization of the scleral spur. All grading of angle metrics was performed by anterior segment OCT graders (HA, KM) at the Doheny Image Reading Center (DIRC). Repeated measures ANOVA and the paired *t*-test were used. Statistical significance was assumed for *p* ≤ 0.05 (version 18.0; SPSS Inc., Armonk, NY).

## Results

The study evaluated fifteen (10 female and 5 male) subjects. The mean age was 31.2 years (range 23–58). The mean values from 30 eyes for the inferior angle metrics 6 o’clock position (IC270), under two different light conditions in the morning and in the afternoon using the Visante TD-OCT are shown in Tables 1 and 2 (Additional file 1; anterior chamber angle metrics). There were statistically significant differences in AOD500 between the condition of pupillary constriction to room light and physiologic mydriasis both in the morning and in the afternoon (*p* < 0.001 for both). AOD750 also showed significant difference for both light conditions in the morning and afternoon (*p* < 0.001 and *p* = 0.031 respectively). Analogously, TISA500 measured under pupillary constriction to room light showed statistically significant difference compared to the condition under physiologic mydriasis in the morning and in the afternoon respectively (*p* < 0.001 and *p* = 0.002). Compared to pupil constriction to light, the mean scleral spur angle increased 9.5 and 12.5% under physiologic mydriasis in the morning and in the afternoon respectively (*p* < 0.001 for both).

**Table 1:** Inferior angle anterior chamber angle metrics under two lighting conditions; with the room lights on and off, from two imaging sessions; morning and afternoon imaging, using the TDOCT

	(LIGHTS)	AOD500 (mm)	AOD750 (mm)	TISA500 (mm^2^)	TISA750 (mm^2^)	SS Angle (^0^)
Morning	IC 270 (ON)	0.48 (0.194-0.776)	0.652 (0.235-1.162)	0.168 (0.079-0.317)	0.309 (0.132-0.558)	42.5 (21.5-57.3)
	IC 270 (OFF)	0.417 (0.068-0.704)	0.57 (0.215-0.975)	0.143 (0.061-0.257)	0.266 (0.096-0.454)	38.8 (7.8-54.6)
*P*-value		<0.001	0.001	<0.001	<0.001	<0.001
Afternoon	IC 270 (ON)	0.474 (0.137-0.751)	0.656 (0.092-1.023)	0.188 (0.065-0.462)	0.321 (0.106-0.50)	42.55 (15.6-56.5)
	IC 270 (OFF)	0.406 (0.091-0.750)	0.592 (0.184-1.146)	0.139 (0.035-0.249)	0.264 (0.078-0.450)	37.82 (10.3-56.3)
*P*-value		<0.001	0.031	0.002	<0.001	<0.001

Mean (minimum and maximum)

Paired *t* test

*P* < 0.05

**Table 2:** Angle parameters from the morning imaging session, with the room lights off, with the angle of incidence at 270° and 5° apart in either direction (265° and 275°)

	IC 270	IC265	IC275	*P* value
AOD500 (mm)	0.417 (0.068-0.704)	0.408 (0.138-0.742)	0.394 (0.143-0.723)	0.81
AOD750 (mm)	0.57 (0.215-0.975)	0.585 (0.042-0.938)	0.535 (0.06-0.931)	0.6
TISA500 (mm^2^)	0.143 (0.061-0.257)	0.138 (0.041-0.233)	0.137 (0.041-0.236)	0.87
TISA750 (mm^2^)	0.266 (0.096-0.454)	0.264 (0.093-0.443)	0.254 (0.067-0.443)	0.86
SS Angle (°)	38.8 (7.8-54.6)	37.9 (15.5-56)	37.1 (16-55.4)	0.8

Mean (minimum and maximum)

*P* < 0.05

The angle of incidence analysis was explored with the lights off in the morning and in the afternoon. The measurements with the angle of incidence offset by 5° toward the nasal and temporal quadrants (IC265-IC275) and at the 6 o’clock position are shown in Table 2. AOD500 and TISA 500 did not show significant differences at IC265 and IC275 compared to the value at IC270 in the morning (*p* = 0.8 and *p* = 0.81 respectively). TISA500 decreased by 3.6 and 4.4% at IC265 and IC275 compared to IC270 in the morning. AOD500 increased by 3.8% at IC265 and decreased by 5.8% at IC275 compared to IC270 in the afternoon respectively but none of these reached the level of significance. AOD750 and TISA 750 did not show significant differences at IC265 and IC275 compared to the value at IC270 in the morning (*p* = 0.6 and *p* = 0.86 respectively).

Finally we assessed the variability of the angle parameters in the morning and in the afternoon at 6 o’clock position (Table 3). There was no statistically significant difference between morning and afternoon measurements for the angle parameters at IC270 (*P* value 0.13-0.93).

**Table 3:** Angle parameters in the morning and afternoon under low light conditions at IC270

Parameters	AM	PM	ABS Diff	% Error	
	Mean ± SD (max)	Mean ± SD (max)	Mean ± SD (max)	Mean ± SD (max)	*P* Value
SS Angle	38.26 ± 9.16 (53.8)	37.82 ± 10.15 (56.3)	37.82 ± 10.15 (56.3)	16.17 ± 20.28 (99.02)	0.72
AOD 500	0.42 ± 0.14 (0.69)	0.41 ± 0.15 (0.75)	0.07 ± 0.06 (0.27)	20.39 ± 22.07 (105.7)	0.68
AOD 750	0.59 ± 0.18 (0.94)	0.6 ± 0.22 (1.15)	0.09 ± 0.09 (0.4)	15.39 ± 14.35 (51.32)	0.68
TISA 500	0.18 ± 0.12 (0.62)	0.14 ± 0.06 (0.25)	0.06 ± 0.11 (0.46)	26.5 ± 33.69 (123.03)	0.13
TISA 750	0.27 ± 0.09 (0.46)	0.27 ± 0.1 (0.46)	0.05 ± 0.04 (0.18)	17.56 ± 18.75 (71.61)	0.93

*AM* Morning

*PM* Afternoon

## Discussion

The current study evaluated the diurnal, lighting, and angle of incidence variation of inferior angle metrics in phakic healthy volunteers using high resolution cross-sectional images of the anterior segment angle with the Visante TD-OCT. Anterior chamber angle metrics did not differ significantly from morning to afternoon imaging, or when the angle of incidence was offset by 5° in either direction away from the inferior angle 6 o’clock position. However, ACA metrics changed when the illumination at the level of the eye changed. Our results support that lightning conditions should be considered when angle imaging is performed, especially with anterior segment OCT, since the illumination conditions can more accurately and easily be controlled.

Physiologic conditions causing dynamic changes in intraocular structures were evaluated before, particularly the response of the iris to light and dark [22]. Quigley et al., using AS-OCT, found a reduction in the iris cross-sectional area after the pupil dilation and they argued that this could be a potential risk factor for angle closure [23]. They hypothesized that eyes with angle closure lose less iris volume on dilation compared to healthy eyes, contributing to iridotrabecular apposition. Additionally in a different study the iris volume was demonstrated to increase after pupil dilation in narrow-angle eyes predisposing to acute angle closure, whereas it was found to decrease in healthy open-angle eyes [24]. In a previous study, we compared the effects of physiologic versus pharmacologic pupil dilation on ACA measurements obtained with spectral domain optical coherence tomography [14]. This study showed a decrease in inferior angle ACA metrics with physiologic pupil dilation under low light conditions and an increase after pharmacologic pupil dilation in normal eyes. These results are in agreement with findings from this study, suggesting that pupil dilation, that happens in dim lighting leads to narrowing of ACA parameters. Therefore, standardizing of lighting conditions is important for objective measurement of ACA metrics.

Studies have already shown that axial length, intraocular pressure and anterior eye biometrics manifest a diurnal rhythm [17, 25, 26]. However, it is not so well understood whether the ACA also shows nyctohemeral changes. In the present study, we compared OCT scan of the ACA between morning and afternoon and found no significant difference.

In addition, results from this paper do not support a statistically significant difference in ACA metrics in open angle eyes when comparing inferior angle scans with differences in the angle of incidence up to 10° apart. This corroborates findings from a previous study, using both time-domain and spectral-domain OCT and might indicate that regional irregularities of the iris profile usually do not play a role in these small variations of the scan position [27]. However, this finding remains to be proven in narrow angles.

Our study is not without limitations. First, we used 30 normal eyes, which is a relatively small population. However, the findings were highly consistent across all eyes in this analysis. Because the scleral spur is better seen on TD-OCT [6, 7] and could be identified in most of the angle scans, it appeared to represent a better anatomical landmark for assessing ACA. In addition, in this study we enrolled only normal eyes to measure ACA metrics and further studies are warranted to investigate these effects on other groups, especially patients with narrow angles. Finally, we chose only the inferior angle for analysis because we assumed that the dynamic ACA metrics is similar in other quadrants. This assumption has been validated in previous studies [14].

## Conclusion

ACA metrics with time domain OCT in normal eyes did not show significant difference from morning to afternoon imaging, or when the angle of incidence was offset by 5° in either direction away from the inferior angle 6 o’clock position. However, there was a significant decrease in angle metrics when the lights were switched off in the imaging room. These finding supports the need to carefully monitor lighting conditions when imaging participants, especially with regards to longitudinal studies.

## Additional file

Additional file 1: Effects of Diurnal, Lighting, and Angle-of-Incidence Variation on Anterior Segment Optical Coherence Tomography Angle Metrics. Description of the data: Angle metrics were recorded from the scans of the morning and the afternoon with different lightining conditions. (XLSX 48 kb)

## Abbreviations

ACA: Anterior chamber angle
AOD: Angle opening distance
AS: Anterior Segment
AZ: Arizona
CA: California
DIRC: Doheny Image Reading Center
FC: Foot candle
IC: Inferior corneal limbus
OCT: Optical Coherence Tomography
PACG: Primary angle closure glaucoma
UBM: Ultrasound Biomicroscopy
TD: Time Domain
TISA: Trabecular-iris space area
